# Supplementary material for: Active site specificity profiling datasets of matrix metalloproteinases (MMPs) 1, 2, 3, 7, 8, 9, 12, 13 and 14
Source: Data Brief. 2016 Feb 22;7:299–310. doi: 10.1016/j.dib.2016.02.036 (PMC4777984; doi:10.1016/j.dib.2016.02.036)
Supplement: Supplementary file 10 — Supplementary material [file mmc10.zip › WebPICS_hMMP9_T_1%/PICS_results.html]

 

PICS results


- # About
- # Manual
- # Analysis
- # Results

- Redraw
- Seqlogo
- Dependency
- Coop

**PICS analysis of protease: hMMP9\_T\_1%**  
138 cleavage sites analyzed (total was 155)  
PICS library made with (T)rypsin, (G)luC or (C)hymotrypsin: T  

|  |  |
| --- | --- |
| Positional occurences    **Values > 10 % are shown** (table for total and relative (in %) values) | Occurences relative to natural abundance   **Values > 2 fold natural abundance are shown** (table) |
|  |  |

  
  
Results (including this html file) in one compressed file for local use.
  
  
Total occurences (open csv)

|  |  |  |  |  |  |  |  |  |  |  |  |  |
| --- | --- | --- | --- | --- | --- | --- | --- | --- | --- | --- | --- | --- |
|  | P6 | P5 | P4 | P3 | P2 | P1 | P1prime | P2prime | P3prime | P4prime | P5prime | P6prime |
| A | 10 | 12 | 11 | 13 | 21 | 28 | 5 | 11 | 31 | 10 | 22 | 13 |
| C | 2 | 4 | 4 | 2 | 2 | 3 | 5 | 5 | 0 | 3 | 1 | 2 |
| D | 3 | 3 | 3 | 0 | 2 | 9 | 0 | 2 | 5 | 8 | 7 | 15 |
| E | 7 | 4 | 5 | 1 | 5 | 10 | 2 | 3 | 3 | 5 | 9 | 9 |
| F | 0 | 8 | 3 | 0 | 6 | 5 | 5 | 6 | 1 | 3 | 4 | 2 |
| G | 0 | 7 | 10 | 2 | 11 | 22 | 1 | 6 | 35 | 17 | 7 | 8 |
| H | 3 | 6 | 5 | 1 | 6 | 4 | 3 | 10 | 6 | 11 | 6 | 4 |
| I | 3 | 4 | 5 | 6 | 3 | 0 | 26 | 15 | 3 | 8 | 9 | 6 |
| K | 0 | 0 | 5 | 0 | 1 | 0 | 2 | 2 | 2 | 3 | 5 | 7 |
| L | 10 | 4 | 11 | 8 | 12 | 2 | 49 | 15 | 2 | 7 | 15 | 8 |
| M | 0 | 2 | 2 | 1 | 9 | 1 | 6 | 8 | 1 | 5 | 6 | 4 |
| N | 4 | 5 | 1 | 1 | 2 | 10 | 2 | 2 | 7 | 10 | 8 | 9 |
| P | 7 | 9 | 9 | 69 | 2 | 12 | 0 | 3 | 2 | 12 | 6 | 19 |
| Q | 3 | 5 | 1 | 2 | 8 | 9 | 9 | 14 | 5 | 5 | 5 | 4 |
| R | 2 | 0 | 3 | 0 | 0 | 0 | 0 | 3 | 1 | 3 | 2 | 1 |
| S | 9 | 5 | 6 | 3 | 18 | 14 | 5 | 6 | 23 | 9 | 7 | 10 |
| T | 5 | 3 | 8 | 2 | 4 | 2 | 1 | 8 | 7 | 5 | 10 | 4 |
| V | 4 | 7 | 9 | 13 | 10 | 0 | 12 | 16 | 4 | 11 | 6 | 10 |
| W | 1 | 0 | 1 | 0 | 1 | 3 | 3 | 1 | 0 | 1 | 0 | 0 |
| Y | 2 | 3 | 3 | 0 | 7 | 1 | 2 | 2 | 0 | 2 | 3 | 3 |

  
  
Relative occurences in % (open csv)

|  |  |  |  |  |  |  |  |  |  |  |  |  |
| --- | --- | --- | --- | --- | --- | --- | --- | --- | --- | --- | --- | --- |
|  | P6 | P5 | P4 | P3 | P2 | P1 | P1prime | P2prime | P3prime | P4prime | P5prime | P6prime |
| A | 7.2 | 8.7 | 8.0 | 9.4 | 15.2 | 20.3 | 3.6 | 8.0 | 22.5 | 7.2 | 15.9 | 9.4 |
| C | 1.4 | 2.9 | 2.9 | 1.4 | 1.4 | 2.2 | 3.6 | 3.6 | 0.0 | 2.2 | 0.7 | 1.4 |
| D | 2.2 | 2.2 | 2.2 | 0.0 | 1.4 | 6.5 | 0.0 | 1.4 | 3.6 | 5.8 | 5.1 | 10.9 |
| E | 5.1 | 2.9 | 3.6 | 0.7 | 3.6 | 7.2 | 1.4 | 2.2 | 2.2 | 3.6 | 6.5 | 6.5 |
| F | 0.0 | 5.8 | 2.2 | 0.0 | 4.3 | 3.6 | 3.6 | 4.3 | 0.7 | 2.2 | 2.9 | 1.4 |
| G | 0.0 | 5.1 | 7.2 | 1.4 | 8.0 | 15.9 | 0.7 | 4.3 | 25.4 | 12.3 | 5.1 | 5.8 |
| H | 2.2 | 4.3 | 3.6 | 0.7 | 4.3 | 2.9 | 2.2 | 7.2 | 4.3 | 8.0 | 4.3 | 2.9 |
| I | 2.2 | 2.9 | 3.6 | 4.3 | 2.2 | 0.0 | 18.8 | 10.9 | 2.2 | 5.8 | 6.5 | 4.3 |
| K | 0.0 | 0.0 | 3.6 | 0.0 | 0.7 | 0.0 | 1.4 | 1.4 | 1.4 | 2.2 | 3.6 | 5.1 |
| L | 7.2 | 2.9 | 8.0 | 5.8 | 8.7 | 1.4 | 35.5 | 10.9 | 1.4 | 5.1 | 10.9 | 5.8 |
| M | 0.0 | 1.4 | 1.4 | 0.7 | 6.5 | 0.7 | 4.3 | 5.8 | 0.7 | 3.6 | 4.3 | 2.9 |
| N | 2.9 | 3.6 | 0.7 | 0.7 | 1.4 | 7.2 | 1.4 | 1.4 | 5.1 | 7.2 | 5.8 | 6.5 |
| P | 5.1 | 6.5 | 6.5 | 50.0 | 1.4 | 8.7 | 0.0 | 2.2 | 1.4 | 8.7 | 4.3 | 13.8 |
| Q | 2.2 | 3.6 | 0.7 | 1.4 | 5.8 | 6.5 | 6.5 | 10.1 | 3.6 | 3.6 | 3.6 | 2.9 |
| R | 1.4 | 0.0 | 2.2 | 0.0 | 0.0 | 0.0 | 0.0 | 2.2 | 0.7 | 2.2 | 1.4 | 0.7 |
| S | 6.5 | 3.6 | 4.3 | 2.2 | 13.0 | 10.1 | 3.6 | 4.3 | 16.7 | 6.5 | 5.1 | 7.2 |
| T | 3.6 | 2.2 | 5.8 | 1.4 | 2.9 | 1.4 | 0.7 | 5.8 | 5.1 | 3.6 | 7.2 | 2.9 |
| V | 2.9 | 5.1 | 6.5 | 9.4 | 7.2 | 0.0 | 8.7 | 11.6 | 2.9 | 8.0 | 4.3 | 7.2 |
| W | 0.7 | 0.0 | 0.7 | 0.0 | 0.7 | 2.2 | 2.2 | 0.7 | 0.0 | 0.7 | 0.0 | 0.0 |
| Y | 1.4 | 2.2 | 2.2 | 0.0 | 5.1 | 0.7 | 1.4 | 1.4 | 0.0 | 1.4 | 2.2 | 2.2 |

  
  
Occurences relative to natural abundance   

|  |  |  |  |  |  |  |  |  |  |  |  |  |
| --- | --- | --- | --- | --- | --- | --- | --- | --- | --- | --- | --- | --- |
|  | P6 | P5 | P4 | P3 | P2 | P1 | P1prime | P2prime | P3prime | P4prime | P5prime | P6prime |
| A | 0.9 | 1.1 | 1.0 | 1.1 | 1.8 | 2.5 | 0.4 | 1.0 | 2.7 | 0.9 | 1.9 | 1.1 |
| C | 1.0 | 2.1 | 2.1 | 1.0 | 1.0 | 1.6 | 2.6 | 2.6 | 0.0 | 1.6 | 0.5 | 1.0 |
| D | 0.4 | 0.4 | 0.4 | 0.0 | 0.3 | 1.2 | 0.0 | 0.3 | 0.7 | 1.1 | 0.9 | 2.0 |
| E | 0.8 | 0.4 | 0.5 | 0.1 | 0.5 | 1.1 | 0.2 | 0.3 | 0.3 | 0.5 | 1.0 | 1.0 |
| F | 0.0 | 1.5 | 0.6 | 0.0 | 1.1 | 0.9 | 0.9 | 1.1 | 0.2 | 0.6 | 0.8 | 0.4 |
| G | 0.0 | 0.7 | 1.0 | 0.2 | 1.1 | 2.2 | 0.1 | 0.6 | 3.6 | 1.7 | 0.7 | 0.8 |
| H | 1.0 | 1.9 | 1.6 | 0.3 | 1.9 | 1.3 | 1.0 | 3.2 | 1.9 | 3.5 | 1.9 | 1.3 |
| I | 0.4 | 0.5 | 0.6 | 0.7 | 0.4 | 0.0 | 3.1 | 1.8 | 0.4 | 1.0 | 1.1 | 0.7 |
| K | 0.0 | 0.0 | 0.6 | 0.0 | 0.1 | 0.0 | 0.2 | 0.2 | 0.2 | 0.4 | 0.6 | 0.9 |
| L | 0.7 | 0.3 | 0.8 | 0.6 | 0.9 | 0.1 | 3.7 | 1.1 | 0.1 | 0.5 | 1.1 | 0.6 |
| M | 0.0 | 0.6 | 0.6 | 0.3 | 2.7 | 0.3 | 1.8 | 2.4 | 0.3 | 1.5 | 1.8 | 1.2 |
| N | 0.7 | 0.9 | 0.2 | 0.2 | 0.3 | 1.8 | 0.3 | 0.3 | 1.3 | 1.8 | 1.4 | 1.6 |
| P | 1.1 | 1.4 | 1.4 | 10.7 | 0.3 | 1.9 | 0.0 | 0.5 | 0.3 | 1.9 | 0.9 | 2.9 |
| Q | 0.6 | 0.9 | 0.2 | 0.4 | 1.5 | 1.6 | 1.6 | 2.6 | 0.9 | 0.9 | 0.9 | 0.7 |
| R | 0.3 | 0.0 | 0.4 | 0.0 | 0.0 | 0.0 | 0.0 | 0.4 | 0.1 | 0.4 | 0.3 | 0.1 |
| S | 1.0 | 0.6 | 0.7 | 0.3 | 2.0 | 1.6 | 0.6 | 0.7 | 2.6 | 1.0 | 0.8 | 1.1 |
| T | 0.7 | 0.4 | 1.1 | 0.3 | 0.5 | 0.3 | 0.1 | 1.1 | 1.0 | 0.7 | 1.4 | 0.5 |
| V | 0.4 | 0.7 | 0.9 | 1.4 | 1.0 | 0.0 | 1.3 | 1.7 | 0.4 | 1.2 | 0.6 | 1.0 |
| W | 0.6 | 0.0 | 0.6 | 0.0 | 0.6 | 2.0 | 2.0 | 0.6 | 0.0 | 0.6 | 0.0 | 0.0 |
| Y | 0.5 | 0.8 | 0.8 | 0.0 | 1.8 | 0.2 | 0.5 | 0.5 | 0.0 | 0.5 | 0.8 | 0.8 |
